# Preservation of the pulmonary branches of the vagus nerve during three-dimensional thoracoscopic radical resection of lung cancer: a retrospective study

**DOI:** 10.1186/s12893-024-02347-w

**Published:** 2024-02-09

**Authors:** Wencong Huang, Jiantian Yang, Huiwen Chen, Peijian Li, Wei Wei

**Affiliations:** 1 Department of Cardiothoracic Surgery, Huizhou Municipal Central Hospital, 41 Eling North Road, Huizhou, 516001 China; 2 Department of Ultrasonic Medicine, Huizhou Municipal Central Hospital, Huizhou, 516001 China

**Keywords:** Three-dimensional thoracoscopy, Pulmonary branches of vagus nerve, Radical resection of lung cancer, Postoperative rehabilitation

## Abstract

**Background:**

In this study, we investigated the effect of preservation of the pulmonary branches of the vagus nerve during systematic dissection of mediastinal lymph nodes, when performing radical resection of lung cancer, on the postoperative complication rate.

**Methods:**

The clinical data for 80 patients who underwent three-dimensional thoracoscopic radical resection of lung cancer in the Department of Thoracic Surgery at Huizhou Municipal Central Hospital between 2020 and 2022 were analyzed. The patients were divided into two groups according to whether the pulmonary branches of the vagus nerve were retained during intraoperative carinal lymph node dissection. The operation time, time until first postoperative defecation, duration for which a chest tube was needed, total chest drainage volume, average pain intensity during the first 5 postoperative days, incidence of postoperative pneumonia, and postoperative length of stay were compared between the two groups.

**Results:**

There was no statistically significant difference in histological staging or in time until first postoperative defecation between the two groups (*p* > 0.05). However, there were significant differences in operation time, the duration for which a chest tube was needed, total chest drainage volume, average pain intensity during the first 5 postoperative days, white blood cell count and procalcitonin level on postoperative days 1 and 5, and postoperative length of stay between the two groups (*p* < 0.05).

**Conclusion:**

Preserving the pulmonary branches of the vagus nerve during carinal lymph node dissection when performing three-dimensional thoracoscopic radical resection of lung cancer can reduce the risk of postoperative complications.

## Background

The vagus nerve is the tenth cranial nerve and contains mixed afferent (sensory), efferent (motor), and parasympathetic nerve fibers that innervate several major organs, including the liver, lungs, spleen, kidneys, and gut. The vagus nerve also plays an important role in regulation of heart rate [1–3]. The pulmonary branches of the vagus nerve is the parasympathetic nerve of the lung and is distributed in the bronchial smooth muscle, glands, and vascular wall. Inferior to the azygos arch, the main branches of the vagus nerve pass posteromedially to the main right bronchus and pulmonary hilum; they then branch off, forming the posterior pulmonary plexus, which contains 77% (62–100%) of the total nerve supply to the right lung [4–6]. Stimulation of the corresponding receptors leads to contraction of bronchial smooth muscle, glandular secretions, vascular congestion, and mucosal swelling, promoting timely expectoration of sputum and preventing postoperative pulmonary infection [7, 8]. Perioperative pulmonary complications have several causes, including damage to the vagus nerve during thoracic surgery, severe pain caused by injury to the intercostal nerve, impaired postoperative lung function, and fear of coughing. Several studies in China and other countries have confirmed that preserving the vagus nerve during radical resection of upper gastrointestinal cancer can effectively reduce postoperative abdominal distention, constipation, vomiting, and other complications [4, 9, 10]. However, few studies have focused on complications after radical resection of lung cancer with preservation of the vagus nerve. In this study, we investigated the effect of preservation of the pulmonary branches of the vagus nerve during systematic dissection of the mediastinal lymph nodes on the postoperative complication rate after radical resection of lung cancer.

## Methods

### Clinical data and grouping

The clinical data for 80 patients who underwent three-dimensional (3D) thoracoscopic radical resection of lung cancer in the Department of Thoracic Surgery at Huizhou Municipal Central Hospital between 2020 and 2022 were analyzed. The patients were divided into two groups: those in whom the pulmonary branches of the vagus nerve was preserved during intraoperative carinal lymph node dissection (group A) and those in whom the pulmonary branches was not preserved (group B).

The inclusion criteria were as follows: age 18–65 years; primary lung cancer requiring radical surgery; and 3D thoracoscopic radical resection of lung cancer performed. The following exclusion criteria were applied: benign pulmonary disease not requiring lymph node dissection; advanced lung cancer requiring only wedge resection; serious underlying disease of the heart, liver, kidney, or other organ affecting postoperative recovery and prolonging the hospital stay; preoperative pulmonary infection or long-term abdominal discomfort, such as abdominal distension or diarrhea; an Eastern Cooperative Oncology Group score of 3 or 4; requirement for conversion to open surgery ; and injury or incision of the pulmonary branches of the vagus nerve during surgery.

### Surgical approach

**Fig. 1:**
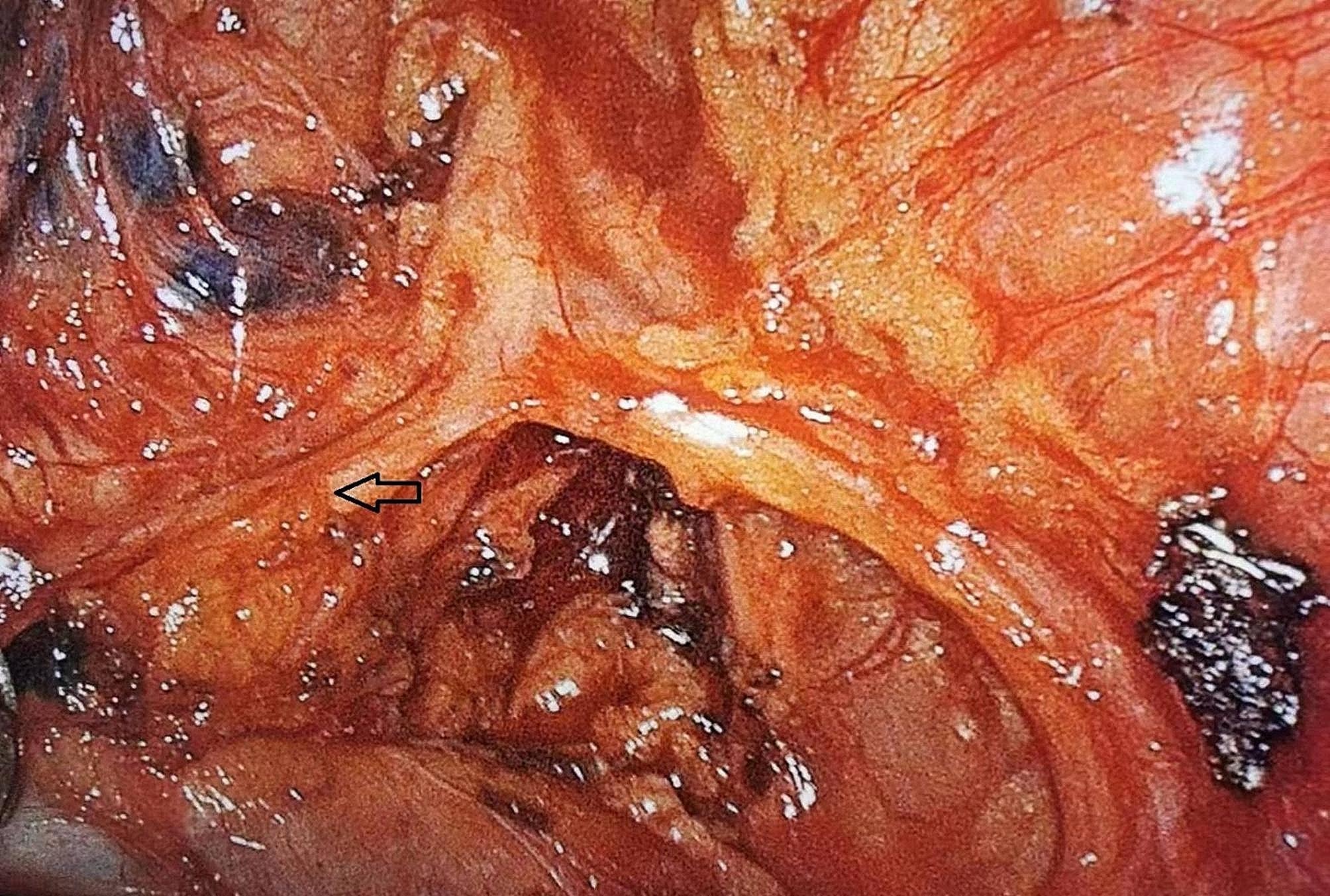
Exposure of the left pulmonary branches of the vagus nerve (arrow) during mediastinal lymph node dissection

**Fig. 2:**
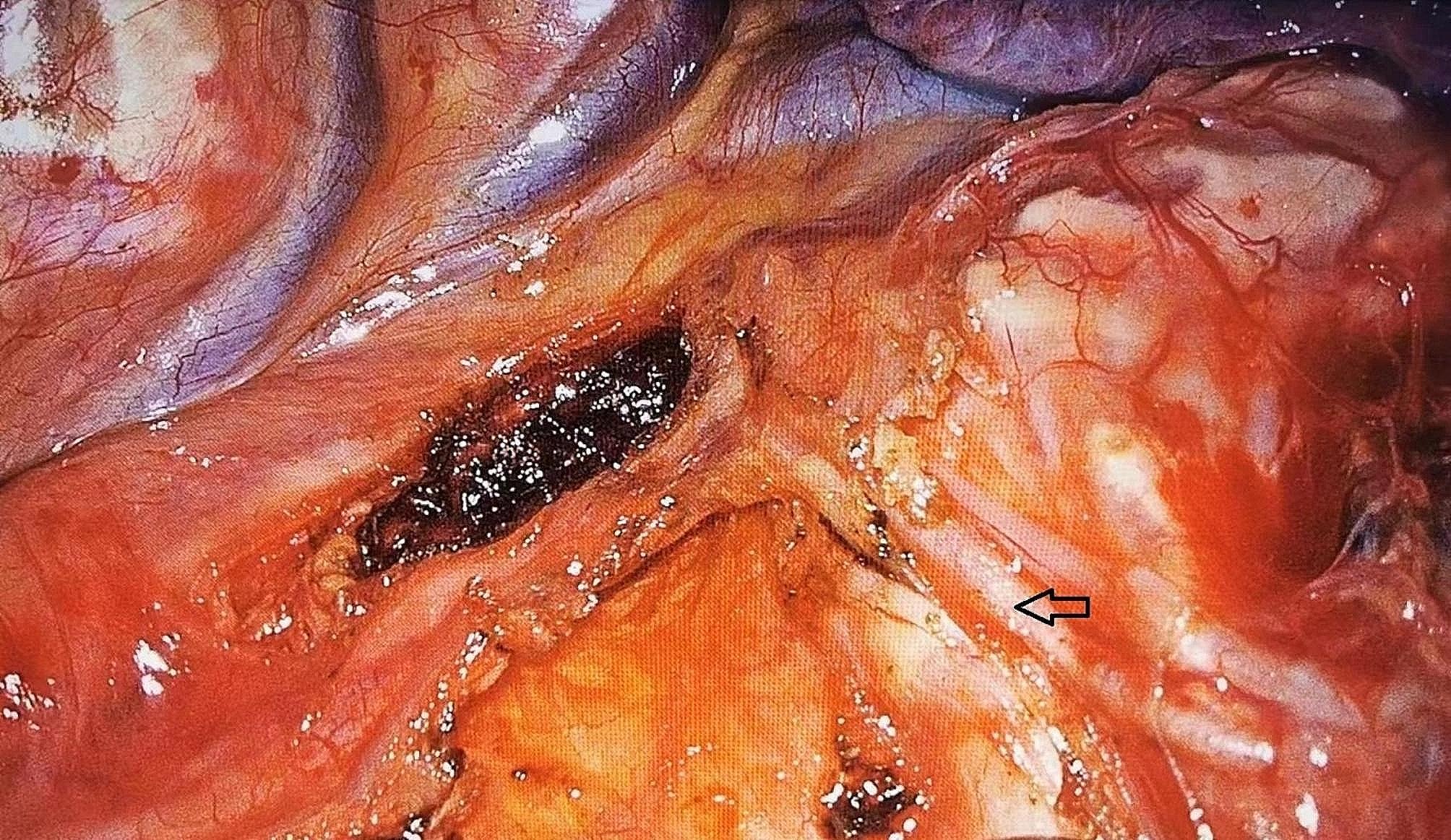
Exposure of the right pulmonary branches of the vagus nerve (arrow) during mediastinal lymph node dissection

Radical surgery for lung cancer was performed using a 3D thoracoscope (Karl Storz SE & Co. KG, Tuttlingen, Germany). Double-lumen endotracheal intubation anesthesia was performed in both groups. Minimally invasive surgery was performed by double-hole thoracoscopy with the patient in the lateral decubitus position. The main surgical access incision was at the level of the third or fourth intercostal space in the midaxillary line and was approximately 3–4 cm in length. The exploratory incision was at the level of the sixth or seventh intercostal space in the axillary line and was approximately 1 cm in length. The thoracoscope and other instruments were inserted, and the pulmonary ligament and mediastinal pleura of the hilum were divided. The pulmonary artery, vein, bronchus, and fissure were completely dissected and transected with a stapler (Endo GIA; Medtronic, Minneapolis, MN, USA), and the hilar mediastinal lymph nodes were systematically dissected. The pulmonary branches of the vagus nerve was either preserved (group A) or not preserved (group B). The exposed left and right pulmonary brancheses of the vagus nerve during mediastinal lymph node dissection are shown in Figs. 1 and 2, respectively. No sealing device was used in either group. Two indwelling #16 chest tubes were placed for postoperative drainage. The chest tubes were removed when chest radiographs showed that the lung had completely reexpansion, no air leak was present, the drainage fluid was a light red or light yellow clear liquid, and the drainage volume was < 200 mL/day.

### Clinical data

The following data were compared between the two groups: operation time, histological stage, time until first postoperative defecation, duration for which a chest tube was needed, total chest drainage volume, average pain intensity during the first 5 postoperative days, incidence of postoperative pneumonia, and postoperative length of stay. The severity of postoperative pain was scored using an 11-point visual analog scale (0, no pain; 10, severe pain). Postoperative pneumonia was diagnosed by radiographic evidence of pulmonary exudate and an increased white blood cell (WBC) count and procalcitonin level in peripheral blood. The WBC count and procalcitonin level were recorded on postoperative days 1 and 5.

### Statistical analysis

Quantitative data are expressed as the mean ± standard deviation and were compared between the two groups using the *t*-test if normally distributed and the rank sum test if not normally distributed. Qualitative data are expressed as the number and percentage and were compared between the two groups using the chi-squared test. All statistical analyses were performed using IBM SPSS Statistics Version 22 (IBM Corp., Armonk, NY, USA). A *p*-value of < 0.05 was considered statistically significant.

## Results

The patients’ demographic and clinical data are shown in Table 1. There was no statistically significant difference in age, sex, weight, preoperative WBC count, histological stage, or time to first postoperative defecation between the two groups (*p* > 0.05). However, there were significant between-group differences in operation time, duration for which a chest tube was needed, total chest drainage volume, average pain intensity during the first 5 days, WBC count and procalcitonin level on postoperative days 1 and 5, and postoperative length of stay (*p* < 0.05).

**Table 1:** Demographic and clinical data for the two study groups

	All patients *n* = 80	Group A *n* = 40	Group B *n* = 40	*p* value
Mean age,years	59.58 ± 10.112	59.05 ± 11.204	60.1 ± 9.001	0.645
Sex (male, %)	36(45%)	17(42.5%)	19(47.5%)	0.653
Mean weights(kg)	59.12 ± 8.991	59.75 ± 7.801	58.49 ± 10.103	0.534
Preoperative WBC(10^9/L)	6.71 ± 2.29	6.49 ± 1.96	6.94 ± 2.59	0.386
Operation time (mean hours ± SD)	3.47 ± 0.782	3.76 ± 0.745	3.19 ± 0.715	0.001
Histological Stage(n,%)				0.599
I A	32(40%)	17(42.5%)	15(37.5%)	
I B	32(40%)	16(40%)	16(40%)	
II B	6(7.5%)	2(5%)	4(10%)	
III A	10(12.5%)	5(12.5%)	5(12.5%)	
Time to first postoperative defecation (mean day ± SD)	2.89 ± 2.365	2.88 ± 1.305	2.9 ± 3.103	0.237
Chest tube duration time(mean day ± SD)	4.7 ± 3.188	3.28 ± 1.502	6.13 ± 3.763	0.001
Total chest drainage volume(ml ± SD)	979.88 ± 678.675	653 ± 289	1306 ± 794	0.001
Average pain intensity during the first 5 days (mean intensity ± SD)	2.06 ± 0.789	1.76 ± 0.682	2.36 ± 0.78	0.001
1st day’s WBC(10^9/L)	16.75 ± 4.146	15.59 ± 3.949	17.91 ± 4.058	0.01
1st day’s PCT(ng/ml)	1.324 ± 3.065	0.502 ± 0.53	2.146 ± 4.167	0.001
5th day’s WBC(10^9/L)	11.33 ± 3.408	9.93 ± 3.176	12.74 ± 3.067	0.001
5th day’s PCT(ng/ml)	0.500 ± 0.670	0.263 ± 0.230	0.737 ± 0.861	0.001
Postoperative length of stay (mean day ± SD)	11.19 ± 4.198	11.7 ± 3.376	10.68 ± 4.875	0.016

PCT, procalcitonin; SD, standard deviation; WBC, white blood cell

## Discussion

In this study, we found a significantly longer mean operation time in group A than in group B despite no significant between-group difference in age, sex, weight, preoperative WBC count, or histological stage. This finding suggests that preserving the pulmonary branches of the vagus nerve during intraoperative dissection of the carinal lymph nodes increases the difficulty of surgery.

The vagus nerve releases acetylcholine (ACh) and promotes contraction of gastrointestinal smooth muscle, resulting in defecation symptoms. Therefore, preserving the vagus nerve during radical resection of a malignant upper gastrointestinal tumor can effectively reduce the likelihood of postoperative abdominal distention and constipation [9]. However, in the present study, we found no significant difference in the time to first postoperative defecation between the two study groups. We attribute this finding to the fact that the patients’ digestive tract symptoms were controlled mainly by the abdominal branch of the vagus nerve; therefore, intraoperative resection of the pulmonary branches of the vagus nerve did not have a significant impact.

There is some evidence indicating that vagotomy can lower the threshold of the nociceptive reflex associated with mechanical retraction, indicating that release of ACh by the vagus nerve can protect against postoperative pain [11]. In our present study, the average pain intensity score was lower in group A than in group B during the first 5 days after surgery, which is consistent with the findings of the previous report [11].

The WBC count and procalcitonin level were lower in group A than in group B on postoperative days 1 and 5, indicating that the vagus nerve plays an important role in the regulation of systemic and local inflammatory responses and can reduce the risk of inflammation [12]. It has been hypothesized that vagal receptors are activated by local proinflammatory factors that transmit signals to the central nervous system through afferent nerves and release ACh through efferent nerves, which are known to produce this neurotransmitter [13]. ACh inhibits the ability of immune cells to produce proinflammatory factors by activating α7 nicotinic ACh receptors (α7nAChR) on these cells [14]. When the vagal circuits are intact, activation of α7nAChR promotes phosphorylation of AKT1 in splenic α7nAChR-expressing CD11b + granulocytes and confines these cells to the spleen. Previous research found that disruption of vagal circuits reduced phosphorylation of AKT1 in splenic α7nAChR + CD11b + granulocytes and facilitated egress of these cells from the spleen to the lung [15]. Moreover, without functional vagal circuits or α7nAChR agonist stimulation, α7nAChR + CD11b + cells accumulated in lungs challenged by lipopolysaccharides or *Escherichia coli*, failed to clear bacteria, and promoted an inflammatory response. ACh also acts on the trachea and bronchial glands, which can result in contraction of bronchial smooth muscle, promote secretion of mucus and timely expectoration, and prevent postoperative accumulation of sputum, thereby reducing the risk of pulmonary infection [7, 8].

The duration for which a chest tube was needed was shorter and the total chest drainage volume was smaller in group A than in group B. We attribute this to the reduction of local inflammation of the lungs and pleura by preservation of the vagus nerve. This decrease in inflammation could reduce the permeability of the pleural capillaries and reduce infiltration of cells, proteins, and fluids from capillaries into the pleural cavity, thereby shortening the length of time for which a postoperative chest tube is needed and reducing the risk of infection in the pleural cavity.

Finally, the postoperative length of stay was shorter in group B than in group A. We consider that the time of discharge from hospital would have been affected by subjective factors, such as the patient’s willingness to be discharged.

## Conclusion

Preserving the pulmonary branches of the vagus nerve during dissection of the carinal lymph nodes when performing 3D thoracoscopic radical resection of lung cancer can reduce the likelihood of postoperative complications. Our study is not without limitations. Retrospective analyses introduce potential bias, so we cannot establish causality. Second, only 80 patients were able to be included in the study, which also limits the power and generalizability of our conclusions.

## Data Availability

The datasets used and/or analyzed during this study are available from the corresponding author on reasonable request.

## Abbreviations

3D: Three-dimensional
WBC: White blood cell
PCT: Procalcitonin
ACh: Acetylcholine
